# Wound Healing Potential of the Salvianolic Acid H and Yunnaneic Acid B—The Rosmarinic Acid Derivatives: Anti-Inflammatory Action and Hemocompatibility In Vitro

**DOI:** 10.3390/molecules31030452

**Published:** 2026-01-28

**Authors:** Oleksandra Liudvytska, Justyna Krzyżanowska-Kowalczyk, Mariusz Kowalczyk, Magdalena Bandyszewska, Weronika Skowrońska, Agnieszka Bazylko, Joanna Kolodziejczyk-Czepas

**Affiliations:** 1 Department of General Biochemistry, Faculty of Biology and Environmental Protection, University of Lodz, 90-236 Lodz, Poland; oleksandra.liudvytska@biol.uni.lodz.pl; 2 Department of Phytochemistry, Institute of Soil Science and Plant Cultivation, State Research Institute, Czartoryskich 8, 24-100 Puławy, Poland; jkrzyzanowska@iung.pulawy.pl (J.K.-K.); mkowalczyk@iung.pulawy.pl (M.K.); 3 Department of Translational Immunology and Experimental Intensive Care, Centre of Postgraduate Medical Education, Marymoncka 99/103, 01-813 Warsaw, Poland; 4 Department of Pharmaceutical Biology, Faculty of Pharmacy, Medical University of Warsaw, Banach a 1, 02-097 Warsaw, Poland; weronika.skowronska@wum.edu.pl (W.S.);

**Keywords:** rosmarinic acid, salvianolic acid H, yunnaneic acid B, plasma, hemostasis, inflammation, inflammasome

## Abstract

Phenolic acids of plant origin are recognized as key bioactive compounds with potential for both internal and topical applications. Although some of these phytochemicals are used for skin care and to improve wound healing, oligomeric derivatives of rosmarinic acid (RA) remain poorly characterized in this context. This study aimed to evaluate the anti-inflammatory potential of salvianolic acid H (SA H) and yunnaneic acid B (YA B) in experimental models related to wound-healing, specifically in skin cells (HaCaT keratinocyte and NHDF fibroblast lines), THP1-ASC-GFP monocytes, and human peripheral blood mononuclear cells (PBMCs). Both SA H and YA B reduced pro-inflammatory cytokine release from HaCaT, NHDF, and PBMCs with efficacy comparable to or exceeding that of RA. Analyses of intracellular pathways of inflammatory response revealed that SA H and YA B were also efficient inhibitors of inflammasome formation in THP1-ASC-GFP reporter cells. Furthermore, SA H showed significant inhibitory effects on the activities of cyclooxygenase-2 and 5-lipoxygenase (IC_50_ = 11.53 µg/mL and 2.41 µg/mL, respectively). None of the examined acids influenced the hemostatic system at concentrations of 1–5 μg/mL. At 50 μg/mL, a slight increase in plasma clotting rate was observed for SA H and RA. These findings indicate that SA H and YA B, two naturally occurring oligomeric derivatives of RA, exert significant anti-inflammatory activity and represent promising agents for further studies on their use to improve wound healing.

## 1. Introduction

Phenolic acids, both benzoic and cinnamic acid derivatives, are a group of compounds widely distributed throughout the plant kingdom. The ongoing growth in interest and progress in research on this class of phytochemicals is driven by their broad range of biological activities and potential applications as pharmaceuticals, health supplements, food additives, and cosmetics [1,2,3]. Currently, particular attention is being paid to the medicinal properties of natural extracts and the biological activity of individual plant metabolites derived from them. They are a key source for modern pharmaceutical and drug discovery, play a significant role in preventing and treating various diseases, and are used in wound healing. In addition, several phenolic acids have proven anti-inflammatory and antimicrobial effects, can permeate the skin barrier, and also have significant potential in the skin care industry due to their antioxidant, photo-protective, anti-wrinkle, or whitening properties.

**Figure 1:**
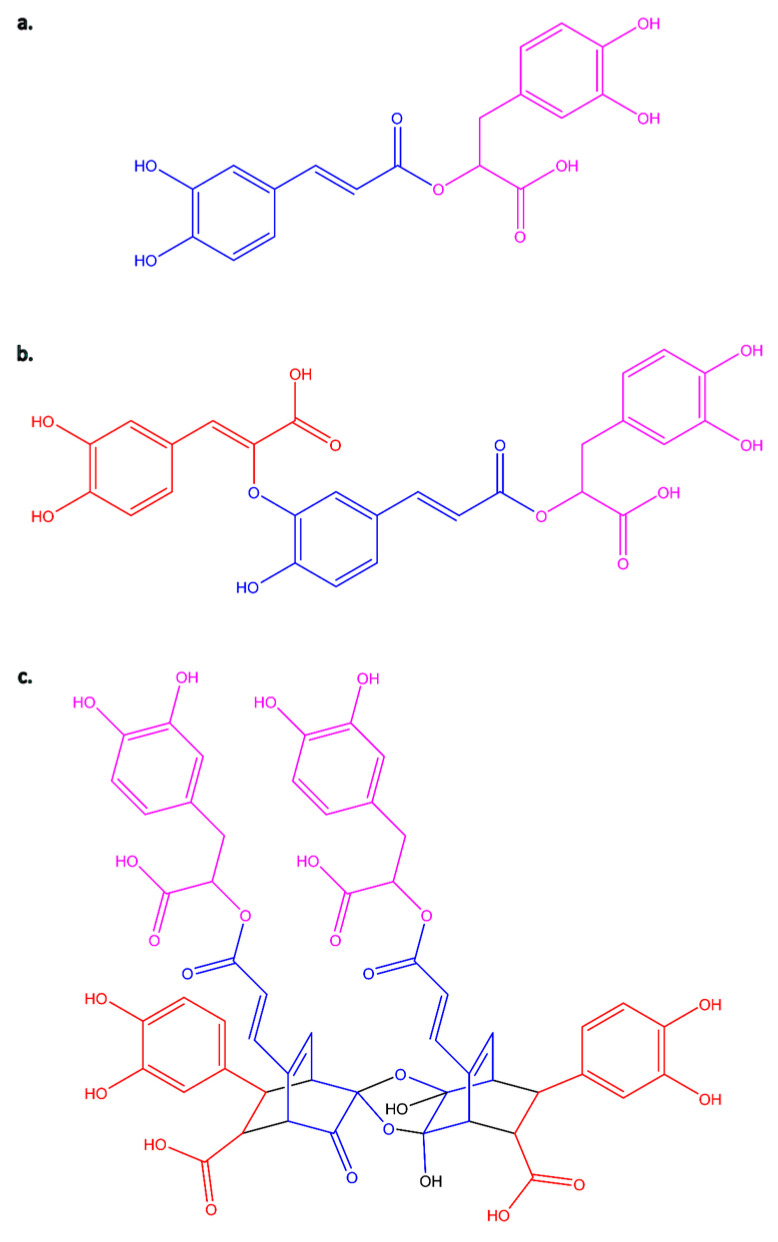
Chemical structure of examined compounds: (**a**) rosmarinic acid (RA) and its derivatives—(**b**) salvianolic acid H (SA H) and (**c**) yunnaneic acid B (YA B). Substructures of rosmarinic acid originating from caffeic acid and 3,4-dihydroxyphenyllactic acid parts are marked in blue and pink, respectively. The additional moieties of caffeic acid (CA) in SA H and YA B are marked in red.

A perfect example of such a bioactive molecule is rosmarinic acid (C_18_H_16_O_8,_ RA, Figure 1a). It is an ester of caffeic acid (3,4-dihydroxycinnamic acid, C_9_H_8_O_4_, CA) and 3,4-dihydroxyphenyllactic acid (also known as α-hydroxyhydrocaffeic acid, salvianic acid A or danshensu, C_9_H_10_O_5_). The biosynthesis of RA involves two parallel pathways: the phenylalanine pathway (for the CA moiety) and the tyrosine pathway (for the phenyl-lactic acid) [4,5]. Its typical sources are plants belonging to the Boraginaceae and Lamiaceae families [5,6]. RA has a wide range of biological activities, including antiviral, antibacterial, antiallergic, and anti-inflammatory properties. Moreover, it also exhibits cardioprotective, hepatoprotective, nephroprotective, anticancer, antidepressant, and antidiabetic activities. Its antioxidant and anti-aging effects have also been demonstrated [7,8,9,10,11,12,13,14,15]. Available literature suggests that RA may also help reverse cancer resistance to first-line chemotherapeutic agents and reduce the toxicity associated with chemotherapy and radiotherapy [16]. This molecule has found applications in various branches of industry, including the pharmaceutical industry, food preservatives [17], and the cosmetics industry as well [18,19,20,21,22,23,24,25].

More importantly, while monomeric phenolic acids such as RA are well studied, increasing evidence suggests that their oligomeric derivatives may exhibit distinct and potentially enhanced biological effects in topical and wound-healing applications. The group of phenolic acids derived from 3,4-dihydroxyphenyllactic acid (danshensu), which combines with caffeic acid in various stoichiometric ratios and bonding arrangements, includes a series of oligomers called salvianolic acids and another one called yunnaneic acids [18,26]. To date, several salvianolic acids (A-K) have been isolated and identified [27,28,29]. Salvianolic acids have been found in various species of the Lamiaceae family, whose representatives are among the most commonly used traditional herbs. A significant source of them is the genus *Salvia*, an important medicinal plant group comprising about 1000 species widely distributed worldwide [30,31]. Salvianolic acids have been shown to have antioxidative, anti-inflammatory, and cardiovascular-protective effects [28,29,32], and their effectiveness in dementia therapy has also been proven [33]. A recently published molecular docking study of salvianolic acids examines their potential as inhibitors and therapeutic agents for the treatment of COVID-19 [34]. Most research on salvianolic acids primarily focuses on salvianolic acid B, also known as lithospermic acid B [35,36,37] or, less commonly used, as monardic acid B [38]. Studies on the biological activities of salvianolic acid A are less common [29,39,40]. Activity data for salvianolic acid C or D are relatively rare, although they are also available [41,42,43,44,45]. The availability of activity data on salvianolic acids is undoubtedly influenced by the fact that these four compounds (salvianolic acid A-D) are dominant in plant extracts and are also commercially available as reference standards. The activity of the remaining salvianolic acids has been little studied, or there is no information on their proven properties. They are often not the primary components of extracts and are not available from commercial sources. Therefore, testing their activity requires a laborious and expensive isolation process. Salvianolic acid H (3′-O-(8″-Z-caffeoyl) rosmarinic acid, C_27_H_22_O_12_, SA H, Figure 1b) is one of such compounds. SA H has been isolated or tentatively characterized in several species of the genera *Salvia*, *Thymus*, *Pulmonaria*, *Lycopus*, *Orthosiphon*, and *Nonea* [46,47,48,49,50,51,52,53,54,55,56,57,58].

Studies on *Salvia yunnanensis* C. H. Wright roots have led to the isolation of eight phenolic acids with even a more complex structural skeleton, namely yunnanic acids A-H [59,60]. In general, yunaneic acids are relatively rare in other plant species. However, some isomers, especially those with lower molecular masses, e.g., D, E, F, have been identified in some species of Boraginaceae and Lamiaceae [61,62,63,64,65,66]. While the high molecular weight molecule, yunnaneic acid B (C_54_H_46_O_25_, YA B, Figure 1c), comprising a bicyclo-[2.2.2]-octene skeleton, originated from an unusual Diels-Alder addition reaction between RA and CA, being a dimer of yunnaneic acid C, is one of the constituents of extracts of *Pulmonaria officinalis* L. [57,67], and *Pulmonaria obscura* L. [67].

Although polyphenol oligomers and polymers have limited oral bioavailability, they can remain highly active in topical applications, particularly in the context of skin disease treatment and promoting wound healing [68,69]. Because of their large molecular mass, phenolic oligomers may interact with cell membranes and membrane proteins, thereby influencing cellular responses through distinct mechanisms or with varying efficacy, compared to the parental compounds. For example, biophysical changes in lipid membranes, such as lipid membrane aggregation and rigidification, were reported for both epigallocatechin-3-gallate, gallocatechin, theaflavin, and theaflavin-3-gallate. However, an increase in the number of hydrophilic side chains (galloyl, hydroxyl, glucoside, gallate) enhanced the reactivity of the examined polyphenols [70]. Significant differences in bioactivity were also observed among procyanidins with distinct degrees of polymerization. Their polymers were more effective at reducing inflammatory cytokine secretion and preserving membrane integrity than mono- and oligomeric procyanidins [71].

Despite applications of some phenolic acids in skin care and wound healing, the biological activities of oligomeric derivatives of RA remain only partly characterized. The present study is the first work to comparatively evaluate the anti-inflammatory potential of SA H and YA B. We hypothesized that SA H and YA B could inhibit inflammatory responses in human in vitro models as effectively as, or even more effectively than their parent compound, RA. Anti-inflammatory effects and cellular safety of SA H and YA B were examined across various in vitro wound-healing models, including skin cell lines, monocytes, and peripheral blood mononuclear cells. Due to the key role of the enzymes of the arachidonic acid metabolism, i.e., cyclooxygenase-2 (COX-2) and 5-lipoxygenase (5-LOX) in the development of inflammatory reactions [72,73], the inhibitory potential of SA H and YA B on these enzymes was also included in this study. The additional goal was to provide data on their impact on the hemostatic system, including the functionality of the plasma coagulation cascade and fibrinolysis. The ability of SA H and YA B to suppress the inflammatory response was assessed relative to the parent compound, RA, a well-known natural anti-inflammatory agent.

## 2. Results

Concentrations of the examined phenolic acids were expressed in micrograms per milliliter to maintain consistency and enable direct comparison with data obtained for *Pulmonaria* extracts in other stages of studies on bioactive components and extracts of these plants. The applied concentrations of 1, 5 and 50 µg/mL correspond to the following micromolar values: 1.9, 9.3 and 92.9 for SA H; 0.9, 4.6 and 45.7 for YA B; 2.8, 13.9, and 138.8 for RA, respectively.

### 2.1. Determination of the COX-2 and 5-LOX Inhibitory Activity

Examination of the COX-2-inhibitory efficacy of the phenolic acids revealed statistically significant effects of both the SA H and YA B. The effect of SA H was dose-dependent, with an IC_50_ value of 11.53 µg/mL (21.4 μM). In contrast, YA B did not exhibit dose-dependency, and its maximal inhibitory effect observed in this assay was approximately 30%. With an inhibitory effect of nearly 55% at a concentration of 1 µg/mL and IC_50_ = 0.91 µg/mL (2.5 μM), RA was the most effective inhibitor of the COX-2 enzyme. In the 5-LOX inhibitor screening, the most significant reduction in enzyme activity was observed with SA H (IC_50_ = 2.41 µg/mL (4.5 μM). The YA B capability of inhibiting the 5-LOX enzyme activity was weaker, and did not exceed 35% (Table 1).

### 2.2. Determination of Anti-Inflammatory Properties of RA and Its Derivatives in Leukocytes—Effects on Pro-Inflammatory Response of Peripheral Blood Mononuclear Cells (PBMCs)

**Figure 2:**
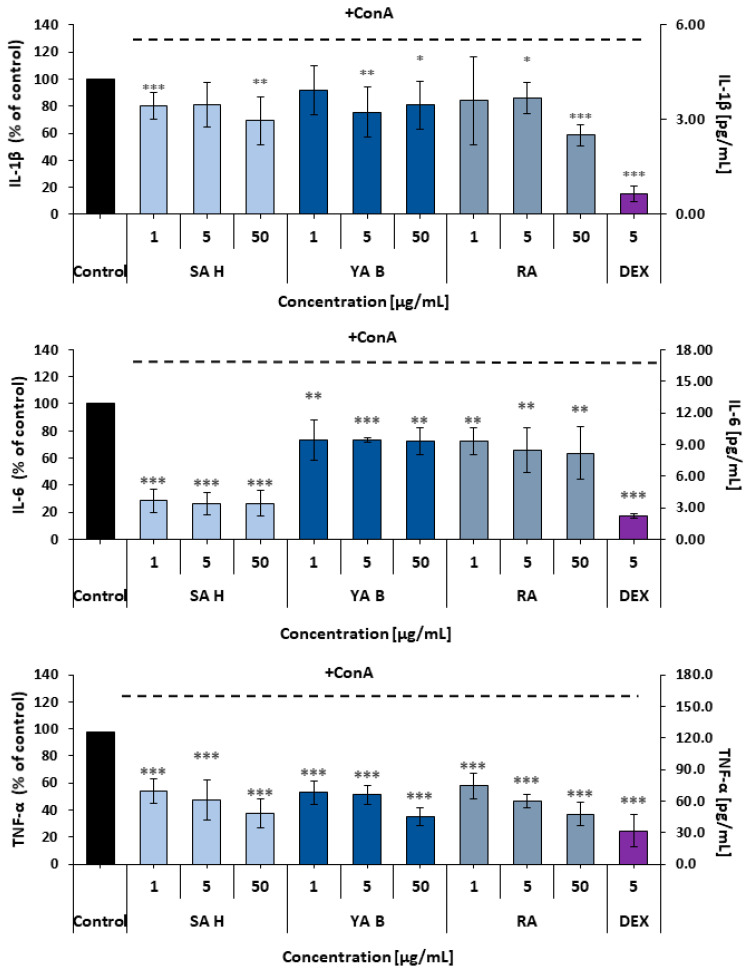
Effects of the examined compounds: SA H, YA B, and RA on pro-inflammatory cytokine release in the concanavalin A (Con A)-stimulated PBMCs. The graphs refer to results from measurements of IL-1β, IL-6, and TNF-α secretion, respectively. DEX (dexamethasone)—a reference compound (an agonist of the glucocorticoid receptor—steroidal anti-inflammatory drug). The cytokine secretion from the Con A-stimulated cells, untreated with the examined phenolic acids, was assumed to be 100%. The figure represents mean values (±SD); * *p* < 0.05, ** *p* < 0.01; *** *p* < 0.001; n = 5.

Effects of the examined phenolic acids on the inflammatory reactivity of PBMCs were determined based on the interleukin 1β (IL-1β), interleukin 6 (IL-6), and tumor necrosis factor alpha (TNF-α) release from these cells. Although all the phenolic acids reduced pro-inflammatory cytokine release, their efficacy varied slightly. The weakest effects on cytokine secretion were observed in IL-1β measurements. In this assay, all examined acids showed comparable efficacy, with maximal reduction in IL-1β release of 30–40%. In the case of IL-6, SA H was the most effective inhibitor (over 70% decrease in IL-6 secretion was observed). The secretion of TNF-α from PBMCs was reduced by all the examined phenolic acids by at least 45–50% (Figure 2).

### 2.3. Effects of the Examined Phenolic Acids on the Inflammasome Formation in THP1-ASC-GFP Cells

**Figure 3:**
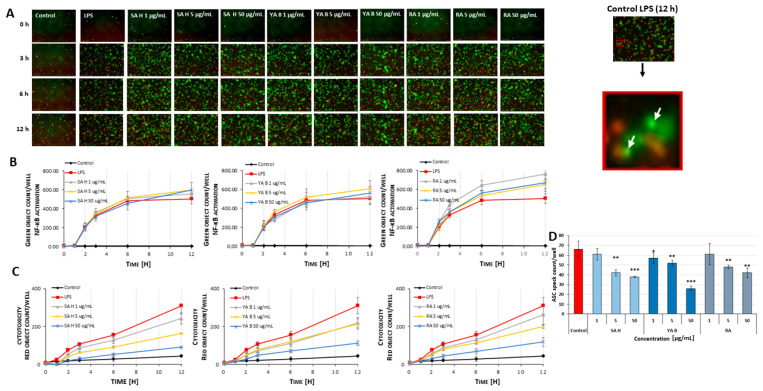
Inhibitory effects of the examined phenolic acids on the LPS-induced formation of ASC specks, determined using the Incucyte SX live-cell analysis system. Panel (**A**): live-cell imaging of THP1-ASC-GFP cells treated with the examined acids: SA H, YA B, and RA (1–50 μg/mL; 24 h.) and stimulated with LPS (1 μg/mL; 3 h.). The scale bar corresponds to 20 µm. Panel (**B**): NF-κB activation in THP1-ASC-GFP cells. Panel (**C**): Quantitative analysis of cell death with DRAQ7. Panel (**D**)**:** The ASC specks number, measured in THP1-ASC-GFP cells, treated as indicated above. Data are shown as mean values (± SD); n = 3. The results represent the average number of specks, based on their counts within the field of view of the visualized cells, recalculated to the cell count in the culture dish (Ø 6.5 mm). The * *p* < 0.05, ** *p* < 0.01, and *** *p* < 0.001 values are related to the cells pre-incubated with the examined phenolic acids (1–50 μg/mL) and stimulated with the LPS, versus the LPS-stimulated cells in the absence of the examined acids. ASC speck formation was observed in real time using the live-cell imaging system Incucyte SX1 software (0–12 h).

The LPS (lipopolysaccharide)-stimulated generation of ASC specks (the apoptosis-associated speck-like protein containing a caspase recruitment domain), determined by live-cell imaging, revealed the anti-inflammatory potential of the examined phenolic acids (Figure 3A). The examined phenolic acids displayed significant (* *p* < 0.05, ** *p* < 0.01, *** *p* < 0.001) inhibitory effects (by approx. 15–60%) on the ASC specks formation in the THP1-ASC-GFP cells (i.e., human leukemia monocytic cells stably expressing the green fluorescent protein tagged ASC), stimulated with LPS (Figure 3D). The most evident reduction in speck generation was observed in cells pre-incubated with YA B and SA H at 50 µg/mL (by about 60% and 43%, respectively). In contrast, in cells pre-incubated with the SA H and RA at a concentration of 1 μg/mL, no significant changes in ASC levels were found.

The release of ASC specks coincided with the number of activated cells, indicating activation of the nuclear factor kappa-light-chain-enhancer of activated B cells (NF-κB). Notable differences in ASC speck release were observed after 12 h of incubation with phenolic acids (Figure 3B). In samples treated with SA H (at conc. of 1, 5, and 50 μg/mL), the highest numbers of activated cells were observed (764 ± 49, 654 ± 47, and 672 ± 94 cells per field of view, respectively), compared to the LPS-stimulated control (approximately 505 ± 54 cells) (Figure 3B). A decrease in cell death was noted in all tested samples (about 15–57%) (Figure 3C).

### 2.4. Effects on the Inflammatory Response of Skin Cells

**Figure 4:**
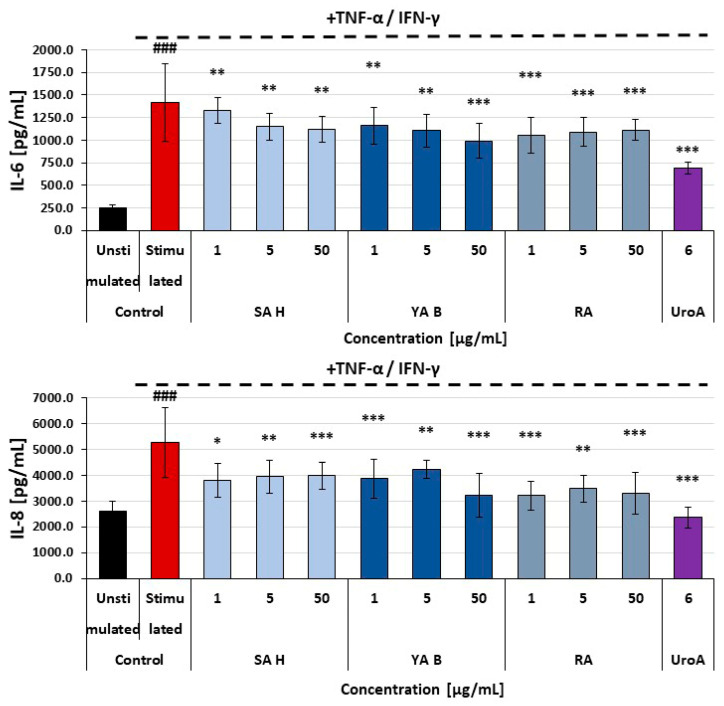
Effects of SA H, YA B, and RA on the secretion of IL-6 and IL-8 by HaCaT cells (keratinocytes). Urolithin A (UroA)—a reference compound (25 µM). The figure represents mean values (±SD); ^###^ *p* < 0.001—unstimulated HaCaT vs. HaCaT treated with TNF-α/interferon-gamma (IFN-γ) in the absence of the examined substances; the cytokine level (pg/mL) detected in samples derived from the TNF-α/IFN-γ -stimulated cells and treated with the examined acids vs. cells treated in the absence of the examined acids: * *p* < 0.05; ** *p* < 0.01; *** *p* < 0.001.

**Figure 5:**
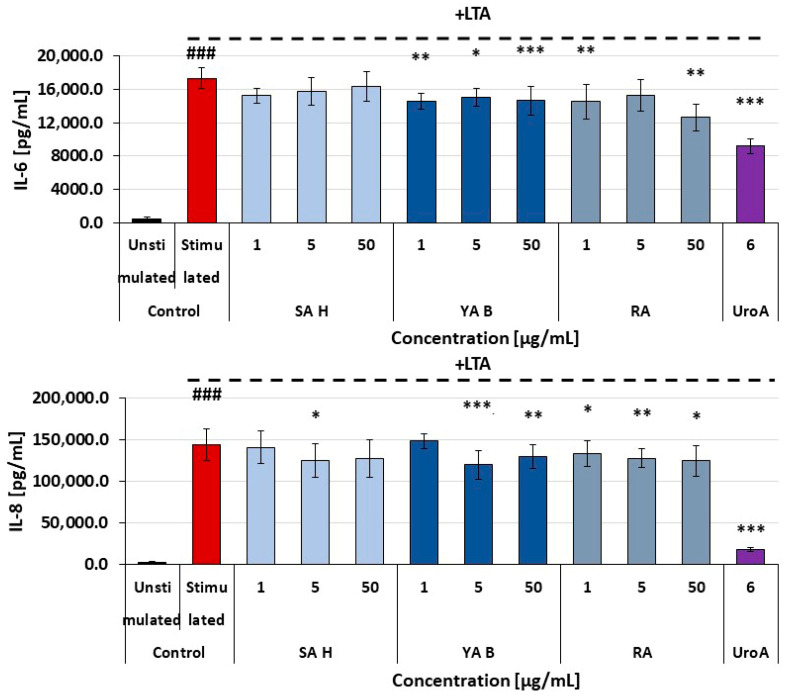
Effects of SA H, YA B, and RA on the secretion of IL-6 and IL-8 by NHDF cells (fibroblasts). Urolithin A (UroA)—a reference compound (25 µM). The figure represents mean values (±SD); ^###^ *p* < 0.001 refers to unstimulated NHDF vs. NHDF treated with TNF-α/IFN-γ in the absence of the examined substances; the cytokine level (pg/mL) detected in samples derived from the TNF-α/IFN-γ—stimulated cells and treated with the examined acids vs. cells treated in the absence of the examined acids: * *p* < 0.05; ** *p* < 0.01; *** *p* < 0.001.

All tested phenolic acids exerted moderate inhibitory effects on the secretion of pro-inflammatory cytokines (i.e., IL-6 and IL-8) in HaCaT cells (human epidermal keratinocyte cell line). The maximal inhibition of cytokine release was about 40%, and no significant differences between the efficacy of SA H, YA B, and RA were found (Figure 4). In normal human dermal fibroblasts (NHDF), the reduction in pro-inflammatory cytokine release was less pronounced, with the highest observed reduction remaining below 25%. Both YA B and RA caused a modest decrease in the release of IL-6 and IL-8 from these cells. In NHDF cells treated with the SA H, a slight decrease in the IL-8 secretion was found, with no effects on IL-6 release (Figure 5).

### 2.5. Effects of the Examined Compounds on HaCaT, NHDF, and PBMCs Viability

The cellular safety of the examined phenolic acids towards skin cells was evaluated using the MTT assay, while their effects on PBMC viability were determined using a resazurin-based metabolic assay. No cytotoxic effects of the examined phenolic acids were found in either HaCaT or NHDF cells (*p* > 0.05). None of the examined phenolic acids influenced the PBMCs’ viability (*p* > 0.05) (Table 2).

### 2.6. Results of the Wound Healing Assay

**Figure 6:**
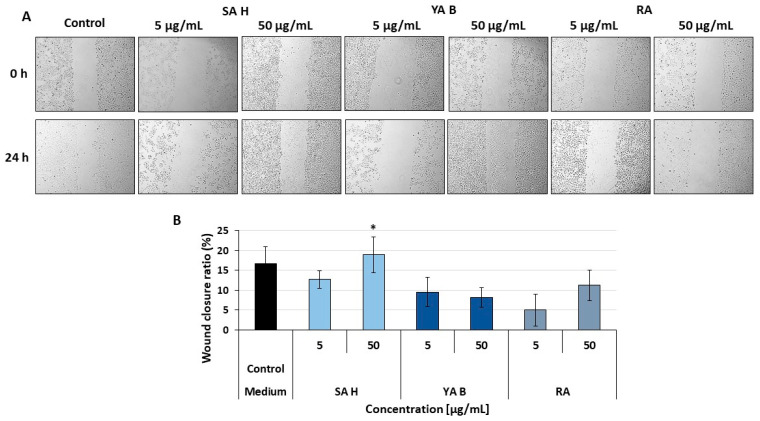
The effect of examined phenolic acids on migrating keratinocytes (HaCaT) to the scratch site. The effects were tested at concentrations of 5 μg/mL and 50 μg/mL. Panel (**A**): representative pictures for each tested group at the test beginning (0 h.) and after 24 h. of incubation, at 4 times magnification. Panel (**B**): the ratio of wound closure. The results are presented as a percentage ratio of the control (untreated with the examined acids). Data are presented as mean ± SD, n = 3; * *p* < 0.05.

In addition to the anti-inflammatory potential, considered one of the key features relevant to the improvement of the healing process, effects of the RA derivatives on skin cell migration were also assessed. A slight, but statistically significant increase in the migration of keratinocytes of the HaCaT cell line to the scratch site compared to the control was observed for the SA H (at a concentration of 50 µg/mL) (Figure 6). The wound closure ratios were 18.96 ± 4.53% in the treated sample, while in the control (untreated) samples, they were 16.69 ± 4.30%. However, this mild enhancement of wound closure suggests a supportive effect rather than a critical contribution of SAH to cell migration into the wound. In contrast, at the same concentration (50 μg/mL), the YA B and RA did not increase cell migration, and the wound closure ratios were 8.15 ± 2.52 and 11.25 ± 3.85%, respectively.

### 2.7. Effect of RA, SA H, YA B on the Hemostasis Efficacy

The hemocompatibility of the examined phenolic acids was evaluated using two different kinetic assays, i.e., the clot formation and fibrinolysis (CFF) assay and the extrinsic coagulation pathway analysis. The biochemical mechanisms of the CFF assay reflect a sequence of key steps in the physiological hemostatic response. The assay initiates with the thrombin-catalyzed conversion of fibrinogen into fibrin. This process continues until the fibrin clot reaches maximal density, resulting in plasma coagulation. The kinetics of this stage are characterized by the maximal velocity of fibrinogen polymerization (V_maxC_, a marker of plasma clotting efficacy). In addition, the sample’s maximal absorbance at 360 nm (A_max_) provides information on fibrin clot density. The second step of the assay enables the examination of the tissue-type plasminogen activator (t-PA)-induced fibrinolytic activity in plasma, leading to proteolytic degradation of the fibrin clot. The fibrinolysis rate is characterized by the V_maxF_ parameter (the maximal velocity of fibrin clot lysis). Results of the CFF assay demonstrated that at concentrations of 1–50 μg/mL, none of the examined phenolic acids influenced the kinetics of blood plasma clotting, the fibrin clot density, or the efficacy of the fibrin degradation (Table 3; *p* > 0.05). The reference anticoagulant drug, argatroban, totally inhibited the coagulation phase when used at a concentration of 5 μg/mL.

**Figure 7:**
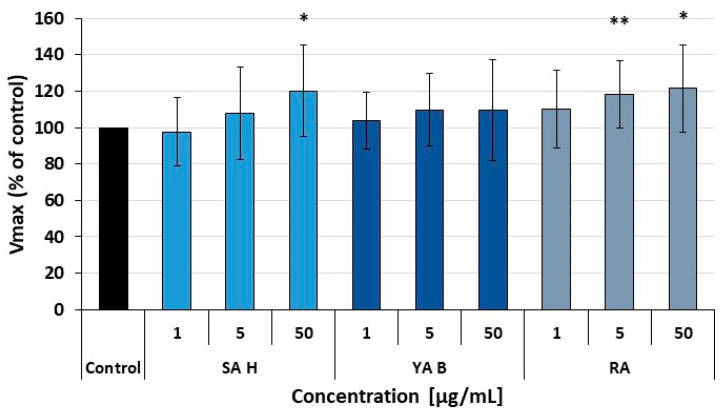
Effects of the phenolic acids on the tissue factor-induced plasma coagulation (the extrinsic pathway of the blood plasma coagulation cascade). The plasma clotting rate was estimated from the maximal velocity (V_max_). The V_max_ values obtained for control samples (plasma untreated with the examined phenolic acids) were assumed to represent 100% of the clotting ability. The results are presented as mean ± SD; n = 5; * *p* < 0.05; ** *p* < 0.01.

Due to the crucial role of the extrinsic coagulation pathway in hemostatic response and wound healing, our hemocompatibility tests also included a preliminary assessment of the effects of RA, SA H, and YA B on the plasma coagulation cascade triggered by the tissue factor (TF). The examined acids did not significantly affect the plasma clotting induced by the TF. However, a slight increase in the plasma clotting rate (the V_max_ parameter) was observed for SA H (at 50 μg/mL) and RA (at 1–50 μg/mL). As the observed enhancement did not exceed 20%, its physiological significance remains to be further investigated. In contrast, YA B did not influence the extrinsic coagulation pathway (Figure 7).

## 3. Discussion

The physiology of inflammation involves a complex interplay of multiple molecular pathways, encompassing three main phases: initiation (1), controlled development (2), and suppression and tissue recovery (3). While a physiological route of the inflammatory response comprising all its characteristic phases is an integral part of the wound healing process, prolonged or chronic inflammation may disrupt healing. A significant role of inflammation in defective wound healing and the development of chronic wounds has been raised in the recent literature [74,75,76]. Excessive and uncontrolled inflammation contributes to tissue damage and delays healing [77]. Moreover, exacerbated and prolonged inflammation not only impairs wound healing but also may increase scarring [78].

The initiation phase of the inflammatory response is primarily driven by activation of components of the hemostatic system, which is subsequently supported by complement cascade proteins and immune cells. The regulation of initial stages and uncontrolled amplification is pivotal to preventing the development of excessive inflammatory response. At the cellular level, these phases are characterized by stimulation of the pattern recognition receptors (PRRs), including Toll-like receptors (TLRs) and nucleotide-binding and oligomerization domain (NOD)-like receptors (NLRs), which activate downstream signaling cascades. A key stage is the activation of the transcription factor NF-κB, which induces the expression of genes for numerous pro-inflammatory mediators, including COX-2 and pro-inflammatory cytokines. Subsequently, the synthesized COX-2 enzyme catalyzes the conversion of membrane-derived arachidonic acid into pro-inflammatory eicosanoids, including prostaglandin E_2_ (PGE_2_). In addition, the inflammatory response is amplified and spread by pro-inflammatory cytokines released from cells (e.g., TNF-α and IL-6) and by the formation of the NLR family pyrin domain-containing 3 (NLRP3) inflammasome [79,80,81,82]. From a pharmacological perspective, each of the aforementioned levels of the inflammatory response (i.e., receptor activation, gene expression, and enzyme activity) may be a target for anti-inflammatory agents. However, in the context of the anti-inflammatory action of plant-derived compounds, their primary molecular targets are enzymes of the arachidonic acid cascade (COX-2, in particular), transcription factors (mainly NF-κB), and some components of the mitogen-activated protein kinase (MAPK)-mediated signaling pathways. Inhibition of COX-2 limits prostaglandin synthesis, thereby attenuating pain and swelling. Simultaneously, suppression of pro-inflammatory cytokines decreases immune cell activation and signaling cascades that propagate inflammation. Furthermore, blocking inflammasome formation (particularly NLRP3 assembly) prevents the activation of caspase-1 and the subsequent maturation of IL-1β and IL-18, reducing the risk of chronic inflammation development. These mechanisms act synergistically, targeting both upstream mediators and downstream effectors to achieve comprehensive anti-inflammatory effects [83,84,85].

Phenolic acids are one of the main classes of plant metabolites, reported to display a wide range of biological activities including antioxidant [86,87], anti-inflammatory [88,89], anti-microbial [90], cardioprotective [91,92], and anti-cancer [93,94] properties in vitro and in vivo. One of the most investigated phenolic acids is RA [95], which in the plant kingdom occurs in free, esterified, glycosidic, and oligomeric forms [96]. RA is commonly found throughout the Boraginaceae and the Nepetoideae subfamily of the Lamiaceae [5,6,97]. Many representatives of those families have been used in traditional folk medicine [98,99], and as culinary herbs, e.g., sage, oregano, thyme, basil, mint, lemon balm, and rosemary [100,101]. The levels of RA in plant extracts can vary widely depending on the species studied, the part of the plant tested, the extraction methods used, or the solvents employed. All this makes comparison of literature data quite complicated. However, its amount ranges from 0.01 to 58.50 mg/g of dry matter [9,97,100,101,102,103,104]. *Pulmonaria officinalis* [57], *Pulmonaria obscura* [67,97], or *Pulmonaria mollis* [66,97] also seems to be a rich source of this compound; its concentration was in the range of 7.00–71.54 mg/g of dry matter. Moreover, RA content, like that of other plant metabolites, is subject to seasonal variation and to differences across phenological stages [105]. Similarly to the fluctuations in RA content in samples of *Thymus* L. species harvested at different ontogenetic stages [106], and comparing the seasons—July vs. October [107], significant differences in SA H [108] as well as YA B [109] content in spring (29.6 ± 9.3 and 216.8 ± 29.3 µg/g DW, respectively) vs. autumn samples (261.9 ± 17.3 and 1834.6 ± 40.5 µg/g DW respectively) were observed in the case of lungwort [57]. RA and its derivatives exhibit diverse and promising biological properties, widely described in numerous reviews and experimental research papers cited in this paper. Therefore, to meet the growing demand for this compound and its various derivatives, several biotechnological methods for its production based on plant tissue culture and metabolic engineering strategies have been developed [110,111,112,113]. Data derived from studies employing diverse experimental systems indicate that RA can suppress inflammation at different molecular levels, including modulation of the *COX2* gene expression [114], inhibition of the COX-2-catalyzed metabolism of the arachidonic acid [115], blockage of the mitogen-activated protein (MAP) kinase-dependent pro-inflammatory pathways [116,117,118], suppression of the NFκB-mediated inflammatory response [119,120] and inhibitory effect on complement cascade activation [121].

RA serves as the core structural unit for a series of more complex oligomeric phenolic acids, including salvianolic and yunnaneic acids (Figure 1). The literature on RA oligomers is rather extensive, reflecting the large number of their naturally occurring derivatives. Many of these compounds are known by different common names, depending on the plant species from which they were first isolated. This nomenclatural variability applies, for example, to several lithospermic, salvianolic, melitric, and monardic acids. Many of these RA-derived molecules are also structural isomers, sharing the same molecular formulas. For instance, salvianolic acids B and E (both C_36_H_30_O_16_) differ in the way their RA and CA residues are linked: salvianolic acid B contains an 8–8′ coupling between CA units, whereas in salvianolic acid E inter-unit connections are different, resulting in a completely different overall conformation. Similarly, lithospermic acid and SA H (both C_27_H_22_O_12_) differ in the arrangement of their phenolic subunits. Although fragmentation mass spectra of the RA-derived compounds often show similarities due to their common structural origin, in many cases, fragmentation patterns observed in MS^2^ spectra are distinct and characteristic, as seen in the examples mentioned above [61,122]. However, it should be pointed out again that differentiation of RA-derived isomeric compounds solely based on MS^2^ spectra is not always feasible.

Contrary to well-established anti-inflammatory effects of RA itself [13,84], the biological properties of its derivatives are less recognized. Studies by Dapkevicius and coworkers revealed high radical-scavenging activity of SA H in the DPPH^•^ (2,2-diphenyl-1-picrylhydrazyl radical)-based assay [47]. Moreover, this molecule is a potential acetylcholinesterase (AChE) inhibitor [123]. SA H and salvianolic acid I, also known as melitric acid A, are regioisomeric compounds [46,124,125,126,127]. Furthermore, their mass spectra fragmentation patterns are nearly identical [61,128]. Therefore, in the characterization of various plant extract components based on HPLC/ESI-MS/MS (high performance liquid chromatography–electrospray ionization tandem mass spectrometry) analyses (metabolic fingerprinting), they are often described as “salvianolic acid H/I”, as distinguishing them with this technique is not possible [61,122,129,130,131,132,133,134,135,136,137,138,139,140,141,142,143]. The same applies to their derivatives, e.g., methyl salvianolate H/I [61,76,137]. Both compounds, salvianolic acid I and H, were isolated from *Salvia cavaleriei* [46]. The comparison of both nuclear magnetic resonance (NMR) spectra is available in Table 9 of the Supplementary Materials of the new review article by Yu and coworkers [26]. In the literature concerning RA derivatives, there is a suggestion that SA H acid may be a transformation product of lycopic acid C. Treatment of lycopic acid C with water was reported to produce SA H. In contrast, methanol treatment produced its methyl ester [53]. Although no other reports have confirmed or observed this conversion, and its isolation and NMR analyses support its native occurrence, the issue may require further, more complete investigation.

In the context of previous research, it seems essential and justified to investigate and determine the biological activities of both SA H and YA B. Our earlier studies on extracts from *P. officinalis* and *P. obscura*, both rich in various RA derivatives, demonstrated their antioxidant and COX-2-inhibitory activity. SA H showed a strong affinity for the COX-2 active site, analogous to the binding mode of indomethacin, as determined in the molecular docking study [58]. We also found that YA B effectively reduced peroxynitrite-induced oxidative and nitrative damage to blood plasma components in vitro [67]. However, the evidence for other biological activities of these compounds remains incomplete.

The present work is the first study to examine the pro-inflammatory potential of YA B and one of the very few on the anti-inflammatory effects of SA H. The studies were conducted to assess their ability to improve wound healing and reduce inflammation in experimental systems of leukocytes and skin cells. We demonstrated the anti-inflammatory potential of the tested phenolic acids and their ability to attenuate the inflammatory response of the aforementioned cells at multiple molecular levels, including decreased secretion of pro-inflammatory cytokines and reduced inflammasome formation. In addition to assessing anti-inflammatory activity, the present study included hemocompatibility tests, comprising a preliminary evaluation of the effects of the examined phenolic acids on functions of the hemostatic system. The hemostatic response begins immediately after an injury and serves not only as a direct defense mechanism to prevent blood loss but also as the first step in the wound healing process [144]. Therefore, the compatibility of tested substances, or their ability to enhance the functions of the hemostatic system, is a key aspect in evaluating the biological effects of materials and compounds intended to suppress inflammation and/or improve wound healing. Overall, the examined phenolic acids demonstrated hemocompatibility. However, SA H showed a minor procoagulant effect at 50 μg/mL, underscoring the need for further in vivo studies to confirm its physiological relevance. Most importantly, the examined RA derivatives showed no cytotoxicity towards either skin cells or PBMCs.

In both skin cell lines and the PBMCs, the inhibitory effects of YA B and SA H on pro-inflammatory cytokine secretion were mainly comparable to the effects observed for the RA (except for the IL-6 release from the Con A-stimulated PBMCs, where the SA H was the most efficient). Notably, the examined acids demonstrated varying anti-inflammatory efficacy against individual cytokines, particularly in assays involving PBMCs. Different abilities of the examined acids to suppress cytokine release may result from distinct molecular pathways regulating these cytokines, and some of these pathways may be more susceptible to modulation by the phytochemicals. For instance, TNF-α synthesis, whose release was most markedly inhibited, depends on the p38 MAPK/NF-κB pathway. In contrast, the synthesis of IL-6 is mediated by the gp130 protein and involves the Janus kinase 1 as well as signal transducers and activators of transcription 1 and 3 (i.e., STAT1 and STAT3) [145]. Furthermore, the Jak/STAT pathway was found to be involved in the regulation of IL-8 transcription in response to pro-inflammatory stimuli [146]. Also, the inhibitory effects of the SA H and YA B on inflammasome formation may be associated with their ability to suppress NF-κB activation. According to the literature, RA, its parental compound, was found to upregulate MAPK/NF-κB-mediated signaling, including inhibition of Erk (extracellular signal-regulated), JNK (c-Jun N-terminal), and p38 MAP kinases, and NF-κB activation [120].

The most common anti-inflammatory strategy is targeting pro-inflammatory enzymes that metabolize arachidonic acid. Arachidonic acid metabolism can be suppressed either by directly inhibiting the catalytic activities of COX-2 and 5-LOX (i.e., at the level of the already synthesized enzymes) or by downregulating the expression of the *COX2* and *ALOX5* genes through modulation of transcription factors such as NF-κB [147,148,149]. When produced, the COX-2- and 5-LOX catalyze the arachidonate metabolism to provide potent mediators that promote the inflammatory response, and their inhibition is considered one of the key steps in anti-inflammatory therapy. Therefore, this study also assessed the inhibitory activity of the examined RA derivatives. Of the two RA derivatives examined, SA H exhibited higher inhibitory potency against COX-2 and 5-LOX, with IC_50_ values of 11.53 and 2.41 μg/mL, respectively. Moreover, its inhibitory effect on the 5-LOX activity was markedly more substantial than that of RA (81.25 µg/mL). This finding is particularly significant, as 5-LOX catalyzes leukotriene biosynthesis and currently available inhibitors of this enzyme, such as zileuton, are associated with adverse effects [150].

The YA-B exhibited weak direct inhibitory effects on COX-2 and 5-LOX activities (at the protein level). However, the activity of these enzymes constitutes only one of the potential targets for the anti-inflammatory action of the RA oligomers. Since the results from experiments on PBMCs indicated that the anti-inflammatory action of YA-B was rather an effect of modulation of the cellular response (i.e., cytokine release and NLRP3 inflammasome formation) than a result of direct inhibition of the COX-2 and 5-LOX enzyme activities, potential effects of the oligomers on *COX2* and *ALOX5* gene expression require further investigation in future studies. Furthermore, it is likely that these compounds can suppress not only NF-κB-dependent signaling (including the expression of pro-inflammatory genes), but also other pro-inflammatory pathways.

In conclusion, the present study provides the first data on the anti-inflammatory activity of SA H and YA B in the context of wound healing, highlighting the promising therapeutic potential of both compounds. In most tests, both examined oligomers displayed anti-inflammatory activity comparable to or greater than that of the parental compound, i.e., RA. However, despite their structural similarities, SA H and YA B appear to share only some molecular pathways and protein targets, particularly regarding their inhibitory effects on cytokine secretion. The efficacy of their anti-inflammatory action also varies depending on the cell type. In addition, in contrast to the YA B, the SA H displayed significant COX-2- and 5-LOX-inhibitory effects. Since finding new, effective inhibitors of this enzyme remains a challenge for modern medicine, results indicating a strong inhibitory effect of SA H on 5-LOX are particularly promising.

Our work may provide a basis for further, more advanced studies on the pharmacological relevance of this oligomer’s anti-inflammatory action. However, as in vitro work, it has significant limitations. While informative, in vitro experimental models do not fully reflect the complexity of wound-healing processes in vivo. Therefore, further studies addressing both anti-inflammatory activity and other aspects relevant to the overall process of wound healing, such as cell migration, tissue regeneration, and long-term safety, are needed. For example, when used at a concentration of 50 μg/mL, neither YA B nor RA revealed promoting effects on cell migration in wound closure tests. However, due to the preliminary character of these tests, this issue requires further studies to determine the effects of both RA and its oligomers on skin cell proliferation and migration, as well as to provide a comprehensive insight into the dose-response. Similarly, further investigations are required to assess the effects of the examined acids on the hemostatic response, including both the coagulation cascade and fibrinolytic proteins. Future research should include in vivo studies to confirm efficacy and safety, explore mechanisms beyond inflammation, and assess formulation strategies for topical applications.

## 4. Materials and Methods

### 4.1. Chemicals

Chemical compounds of the article: Rosmarinic acid (PubChem CID: 5281792); Salvianolic acid H (PubChem CID: 10052949); Yunnaneic acid B (PubChem CID: 10558114).

DMEM high glucose w/stable glutamine w/sodium pyruvate, RPMI1640 1640 w/stable glutamine w/25 mM HEPES, Lymphosep, penicillin-streptomycin solution, Dulbecco’s phosphate-buffered saline (DPBS) without Ca^2+^ and Mg^2+^, fetal bovine serum (FBS), and trypsin-EDTA solution were purchased from BioWest (Nuaillé, France). Trypan blue solution was purchased from BioRad (Hercules, CA, USA). Dimethyl sulfoxide (DMSO, 99.8%, HPLC grade), ethyl acetate, methanol, and *n*-butanol were purchased from POCh (Gliwice, Poland). Concanavalin A, MTT (3-(4,5-dimethylthiazol-2-yl)-2,5-diphenyltetrazolium bromide), In Vitro Toxicology Assay Kit, Resazurin based (TOX8-1KT), and thrombin were purchased from Sigma-Aldrich (St. Louis, MO, USA). The Dia-PT reagent was purchased from Diagon (Budapest, Hungary), and the Actilyse^®^ fibrinolytic reagent (the tissue-type plasminogen activator) was from Boehringer Ingelheim (Ingelheim am Rhein, Germany). Recombinant human interferon-gamma (IFN-γ), recombinant human tumor necrosis factor-alpha (TNF-α), and lipoteichoic acid (LTA) from Gram-positive bacteria *Staphylococcus aureus* were purchased from InvivoGen (Toulouse, France). Kits for the fluorometric screening of potential COX-2 and 5-LOX inhibitors were purchased from Abcam (Cambridge, UK). The enzyme-linked immunosorbent assay (ELISA) antibody sets for interleukins and TNF-α, and the Reagent Set B, were purchased from BD Biosciences (Franklin Lakes, NY, USA).

### 4.2. Isolation of RA and Its Derivatives

The phenolic acids studied were isolated and analyzed using high-resolution mass spectrometry and one-dimensional ^1^H and ^13^C NMR spectroscopy, according to the procedures described in our previous publications [57,67]. *Pulmonaria officinalis* L. grounded aerial parts were defatted with chloroform and afterward extracted with a mixture of water–methanol (80:20). After filtration, the crude extract was evaporated under vacuum and freeze-dried. Next, it was purified stepwise using different chromatographic methods. First, the extract was applied to a preconditioned RP-C18 column (80 × 100 mm, Cosmosil 140 C_18_-PREP, 140 µm; Nacalai Tesque, Inc., Kyoto, Japan), followed by removal of polar constituents (1% MeOH *v*/*v*), while the phenolic-rich fraction was eluted with 50% methanol. In the next step, the enriched phenolic fraction was fractionated by low-pressure chromatography on a Sephadex LH-20 (Sigma-Aldrich, Steinheim, Germany) column (48 × 400 mm) using a gradient of methanol (5–100%, *v*/*v*). Obtained/collected fractions (FR0–FR10) were subsequently subjected to a reversed-phase column (32 × 300 mm, Cosmosil 40C18-PREP, 40 µm; Nacalai Tesque, Inc., Kyoto, Japan), which yielded several sub-fractions. The composition of the fraction and subfractions was monitored by LC-MS, as described previously [57]. RA, SA H, and YA B were further purified by a semi-preparative HPLC using a Gilson chromatographic system (Gilson Inc., Middleton, WI, USA) equipped with an evaporative light scattering detector (ESLD, Gilson PrepELS II), on the Atlantis T3 Prep OBD column (10 × 250 mm, 5 µm, Waters, Milford, MA, USA) [57,67].

The purity of the isolated compounds was determined by isocratic ultra-high-performance liquid chromatography (UHPLC) with charged aerosol (CAD) and high-resolution mass spectrometry detection. Purity was determined as 98%, 97%, and 93% for RA, SA H, and YA B, respectively.

### 4.3. Cyclooxygenase-2 (COX-2) and 5-Lipoxygenase (5-LOX) Inhibitor Screening

COX-2 and 5-LOX activities were determined according to the manufacturer’s protocols (Abcam, Cambridge, UK), using indomethacin and zileuton as reference inhibitors. The COX-2 Inhibitor Screening Kit (Fluorometric) (Abcam, Cambridge, UK) enables measurements of the peroxidase component of COXs. The assay is based on a fluorometric detection of prostaglandin G_2_, an intermediate product generated by the COX enzyme, whose presence is determined at λ_Ex/Em_ of 535/587 nm.

5-Lipoxygenase Inhibitor Screening Kit (Fluorometric) (Abcam, Cambridge, UK) detects hydroperoxides generated during the 5-LOX-catalyzed lipoxygenation reaction at λ_Ex/Em_ of 500/536 nm. As a reference LOX inhibitor (a positive control), Zileuton (0.25 μg/mL) was used.

### 4.4. Cell Cultures

The experimental concentration range of phenolic acids (1–50 μg/mL) was established based on our previous investigations on the bioactivity of *Pulmonaria* extracts [63] and the antioxidant properties of YA B [67], as well as literature data [151]. According to the literature, plasma concentrations of phytochemicals or their metabolites typically range from nanomolar to a few micromolar. For example, the blood plasma concentration of CA ranges from 0.45 to 1.35 μM [151].

Due to their pivotal role of leukocytes and skin cells in inflammatory processes and wound healing, various leukocyte populations (peripheral blood mononuclear cells and monocytes) and two distinct skin cell lines were employed as cellular experimental models in this study.

THP1-ASC-GFP cells were cultured in RPMI-1640 medium containing 2 mM L-glutamine, 25 mM HEPES, phenol red (Gibco, Laboratories, Grand Island, NY, USA), supplemented with 10% FBS (heat-inactivated) (EURx, Gdansk, Poland), 100 μg/mL Normocin™ (InvivoGen, San Diego, CA, USA), and Pen-Strep (100 U/mL–100 μg/mL) (Gibco, Laboratories, Grand Island, NY, USA).

Human keratinocytes (HaCaT) cell line and normal human dermal fibroblasts (NHDF) cell line (both purchased from the Lonza Group Ltd., Basel, Switzerland) were cultured in DMEM high glucose medium (w/L-glutamine, w/sodium pyruvate), supplemented with 10% FBS and 1% penicillin and streptomycin solution, in a humidified incubator, at 37 °C and 5% CO_2_.

Human peripheral blood mononuclear cells (PBMCs) were isolated from buffy coats, purchased from the Regional Center of Blood Donation and Blood Treatment in Lodz, Poland, as anonymized material. Studies on the biological activity of the three examined phenolic acids were approved by the Committee on the Ethics of Research at the University of Lodz, Poland (9/KBBN-UŁ/II/2013). The isolation procedure was performed according to our previously described protocol [152], using the Lymphosep medium (BioWest, Nuaillé, France). Cell count and viability were measured using an automatic cell counter (BioRad, Hercules, CA, USA) based on trypan blue staining [153]. After the isolation, PBMCs (1.5 × 10^6^ cells/mL, suspended in RPMI-1640 medium + 10% FBS + 0.1% of penicillin-streptomycin) were seeded onto 96-well microplates (3.75 × 10^5^ cells/well) and pre-incubated (1 h) with the examined phenolic acids (at final conc. of 1–50 µg/mL, in a laboratory CO_2_ incubator, at 37 °C and 95% humidity). After the pre-incubation, the PBMCs pro-inflammatory response was induced by Con A solution (added to the final conc. of 10 µg/mL). The cells were then cultured for 24 h and centrifuged to obtain the supernatant (cell culture medium).

### 4.5. Determination of Anti-Inflammatory Effects of RA and Its Derivatives in the THP1-ASC-GFP Cells

THP1-ASC-GFP cells were seeded onto black 96-well plates with clear bottoms at a density of 30 × 10^3^ cells/well. After 3 h, the growth medium was exchanged for phenol red-free RPMI-1640. After 24 h of culturing, the cells were pre-incubated for another 24 h, with the examined phenolic acids (at concentrations of 1–50 μg/mL), and then, activated by the lipopolysaccharide—LPS (O55:B5, from *Escherichia coli*, Sigma-Aldrich, St. Louis, MO, USA; L6529; at the final conc. of 1 µg/mL, for 3 h). Formation of the ASC-GFP specks was analyzed in real time (0–12 h) using the Incucyte SX1 imaging system (Sartorius, Göttingen, Germany).

### 4.6. Studies Employing Human Peripheral Blood Mononuclear Cells (PBMCs)

#### 4.6.1. Measurements of Pro-Inflammatory Cytokine Release

IL-1β, IL-6, and TNF-α release was quantified in cell culture medium supernatants, using the enzyme-linked immunosorbent assay (ELISA), i.e., BD OptEIA^TM^, according to the manufacturer’s instructions (BD Biosciences, Franklin Lakes, NY, USA). The used assay kit components included: the Reagent Set B (Cat. #550534), Human IL-1β ELISA Set (Cat. #557953), Human IL-6 ELISA Set (Cat. #555220), and Human TNF ELISA Set (Cat. #555212).

The pro-inflammatory response of PBMCs was induced using Con A, selected based on previously optimized protocols [147]. Con A is a well-established T-cell activator and inducer of inflammatory responses in experimental models, including studies on the anti-inflammatory activity of plant-derived compounds. Considering the involvement of T lymphocytes in skin regeneration and wound healing [154], using Con A in experimental systems reflects immune mechanisms relevant to tissue repair phases, where T-cell-mediated signaling plays a key role.

#### 4.6.2. Cytotoxicity Tests

The isolated PBMCs (1.5 × 10^6^ cells/mL) were treated with the phenolic acids (1–50 μg/mL) and cultured in 96-well microplates (3.75 × 10^5^ cells/well), as described above. The control samples (assumed to have 100% of cell viability) were the cells untreated with the examined phenolic acids. The reference wells with 0% cell viability contained PBMCs treated with 0.5% Triton X-100, a cell-lytic reagent. Cell viability was estimated after 24 h of PBMC culture with the examined phenolic acids, using the resazurin solution (In Vitro Toxicology Assay Kit, Resazurin-based) (Sigma-Aldrich, St. Louis, MO, USA). Cell viability was determined in a microplate spectrophotometer, BMG Labtech SpectroStarNano (BMG LabTech, Ortenberg, Germany) (λ = 600 nm; a reference wavelength: 690 nm), after 4 h. of incubation with resazurin [155].

### 4.7. Studies Employing Skin Cell Lines (HaCaT and NHDF Cells)

#### 4.7.1. HaCaT Cells Stimulated with TNF-α and IFN-γ

The HaCaT cells (12.5 × 10^3^ cells/mL) were grown to reach 80% confluency (i.e., 48 h) in 24-well plates. Then, the medium was aspirated, the cells were rinsed with DPBS, and the examined phenolic acids (suspended in fresh cell culture medium) were added to the wells. After a 4 h incubation, the cells were stimulated with a mixture of TNF-α and IFN-γ (10 ng/mL in DPBS). After another 24 h. of incubation, supernatants were collected and stored at −20 °C until assayed. A non-stimulated control samples was cells untreated with the examined phenolic acids.

#### 4.7.2. NHDF Cells Stimulated with Lipoteichoic Acid (LTA)

The NHDF cells (1/3 of the bottle at 80% confluency) were seeded in 24-well plates and incubated to reach 80% confluency (i.e., 5–7 days). Then, the culture medium was gently aspirated, and the cells were washed with DPBS. Subsequently, the medium with the dissolved examined substances was applied, followed by a treatment with a solution of LTA (10 μg/mL, in DPBS). To the non-stimulated control wells, instead of LTA, DPBS was added. Supernatants were collected after 24 h. of incubation and stored at −20 °C until assayed.

#### 4.7.3. Measurements of IL-6 and IL-8 Secretion from HaCaT and NHDF Cells

The cytokine levels were quantified in supernatants collected from HaCaT and NHDF cell cultures, stimulated with TNF-α and IFN-γ (HaCaT keratinocytes) and LTA (NHDF fibroblasts), using ELISA tests (BD Biosciences, San Diego, CA, USA). Urolithin A (UroA), used at a concentration of 25 μM (in the amount of 6 μg/mL) was used as a reference inhibitor of IL-6 and IL-8 secretion by skin cells [156].

#### 4.7.4. Wound Healing Assay

The HaCaT cells (1.75 × 10^5^ cells/mL) were grown in 12-well plates until a confluent monolayer was formed (24 h). The FBS-enriched medium was replaced with FBS-free medium, and the cells were incubated for another 4 h. Subsequently, the single-cell layer was scratched with a 200 μL pipette tip, the medium was aspirated, and the cells were washed with DPBS. Then, the FBS-free medium containing the examined phenolic acids at 5 and 50 μg/mL was added to the cells. Photos were taken using the microscope with a Nikon camera (Nikon Eclipse TS100, Nikon Instruments Inc., Melville, NY, USA, supported by NIS-Elements BR 3.22 Software) immediately after the compounds had been applied and 24 h later. The results were presented as wound-closure ratios. For this purpose, the surface area of the scratch at 0 h and after 24 h of incubation with the tested samples was compared. The control sample was the FBS-free medium.

#### 4.7.5. Cytotoxicity Assays in HaCaT and NHDF Cells

Effects of the examined phenolic acids on HaCaT and NHDF viability were determined using the 3-[4,5-dimethylthiazol-2-yl]-2,5-diphenyl tetrazolium bromide (MTT) metabolic assay. The HaCaT (12.5 × 10^3^ cells/mL) and NHDF (1/3 of the bottle with 80% confluence on the plate) cells were cultured in 24-well plates for 48–72 h and 5–7 days, respectively, to reach the 80–100% confluence degree. The examined phenolic acids were then added (to a final conc. of 1–50 μg/mL) and incubated for 24 h. Subsequently, the medium was aspirated, the cells were rinsed with DPBS, and a freshly prepared MTT solution (0.5 mg/mL, suspended in DMEM high glucose) was added. The cells were then incubated with the stain for 1 h. Next, the medium was aspirated. DMSO solution was added to each well, and the mixture was shaken for 10 min. The absorbance was measured at 570 nm, using a correction path of 630 nm [157].

### 4.8. Effects of the RA, SA H, and YA B on the Hemostatic System

To obtain a general insight into the interactions of the examined phenolic acids with plasma components of the hemostatic system, the clot formation and fibrinolysis (CFF) assay was applied [158]. Measurements were performed in 96-well plates using a SpectrostarNano microplate spectrophotometer at 360 nm. The working reagent was composed of 0.05 M tris-buffered saline (pH 7.4), the thrombin enzyme (0.75 U/mL), the t-PA (0.264 ng/mL), and 7.5 mM CaCl_2_.

Since the physiological plasma hemostatic response to skin injury is primarily dependent on the tissue factor-triggered initiation of the blood coagulation cascade, the effects of the examined phenolic acids on the extrinsic pathway of plasma coagulation were also determined. Plasma clotting was initiated with the Dia-PT reagent (Diagon, Budapest, Hungary), which contains tissue factor, lipids, and calcium ions. Kinetics measurements were conducted at λ = 360 nm, in 96-well microplates (in the SpectrostarNano microplate spectrophotometer—BMG LabTech, Ortenberg, Germany). The reagent mixture contained 50 μL of human plasma (native or preincubated with the phenolic acids), 250 μL of tris-buffered saline supplemented with calcium ions (0.05 M Tris, 0.9% NaCl, 0.025 M CaCl_2_, pH 7.4), and 5 μL of the Dia-PT reagent. The DiaPT reagent was added to the microplate wells last, immediately before starting measurements [159].

### 4.9. Statistical Analysis

The statistical analysis was performed using the STATISTICA 13.0 PL software (StatSoft Inc., Tulsa, OK, USA). The first analysis step was to eliminate the uncertain data using Grubbs’ test (GraphPad Prism 5.01, San Diego, CA, USA). Next, the differences between groups were assessed using the nonparametric Wilcoxon test (for unpaired data), and the *t*-test was used for normally distributed data. The *p* < 0.05 level was considered statistically significant. All the values in this work are expressed as mean ± standard deviation (±SD).

## Figures and Tables

**Table 1:** The COX-2 and 5-LOX-inhibitory efficacy of SA H, YA B, and RA. Results for the reference compounds are presented as % of inhibition: indomethacin, a reference COX inhibitor, at a concentration of 5 µg/mL (14.0 µM); zileuton, a reference LOX inhibitor, at a concentration of 0.25 µg/mL (1.1 µM); n = 5.

	Salvianolic Acid H	Yunnaneic Acid B	Rosmarinic Acid	Indomethacin	Zileuton
	IC_50_			% of Inhibition	
COX-2 inhibition	11.53 µg/mL (21.4 μM)	>100 µg/mL * (91.3 μM)	0.91 µg/mL (2.5 μM)	93.66 ± 0.36	-
5-LOX inhibition	2.41 µg/mL (4.5 μM)	>100 µg/mL * (91.3 μM)	81.25 µg/mL (225.5 μM)	-	32.93 ± 3.11

* The IC_50_ value estimation was limited by the weak inhibitory activity against 5-LOX and interference caused by higher concentrations of YA B during the assay.

**Table 2:** The cellular safety of RA, SA H, and YA B. The cell viability was measured after 24 h of incubation with the acids. In HaCaT and NHDF cell lines, the MTT (3-(4,5-dimethylthiazol-2-yl)-2,5-diphenyltetrazolium bromide) assay was used, and results (mean ± SD) were derived from 3 independent experiments. PBMCs’ viability was determined using the resazurin-based metabolic test, and data (mean ± SD) were derived from 8 independent experiments. The control (untreated) cells’ viability was assumed as 100%; *p* > 0.05.

Type of Sample	[μg/mL]	HaCaT Viability (%)	NHDF Viability (%)	PBMCs Viability (%)
Control (cells untreated with the acids)	0	100.00 ± 0.00		
Salvianolic acid H	1	102.52 ± 3.39	95.92 ± 5.51	102.77 ± 7.00
	5	100.28 ± 2.57	97.74 ± 4.99	96.91 ± 5.99
	50	104.08 ± 7.03	98.33 ± 7.96	107.23 ± 7.95
Yunnaneic acid B	1	100.63 ± 3.11	96.13 ± 8.56	97.52 ± 13.45
	5	98.62 ± 5.97	98.58 ± 7.92	95.29 ± 16.26
	50	101.82 ± 4.51	99.29 ± 5.81	98.49 ± 10.98
Rosmarinic acid	1	99.57 ± 6.05	96.47 ± 3.73	92.08 ± 11.60
	5	100.67 ± 5.04	98.05 ± 4.06	91.40 ± 12.84
	50	99.10 ± 9.66	102.91 ± 5.56	93.79 ± 10.85

**Table 3:** Determination of RA and its derivatives on the clot formation and fibrinolysis efficacy in human blood plasma, determined in the CFF assay. The hemostatic activity of the control plasma (untreated with the examined phenolic acids) was assumed as 100%. The results are presented as mean ± SD; n = 6, *p* > 0.05.

Type of Plasma Sample	[μg/mL]	V_maxC_ (%)	A_max_ (%)	V_maxF_ (%)
Control	0	100.00 ± 0.00		
Salvianolic acid H	1	106.47 ± 10.51	106.19 ± 8.16	101.86 ± 9.15
	5	103.10 ± 5.35	104.65 ± 5.54	100.75 ± 7.30
	50	100.60 ± 3.80	102.78 ± 4.73	99.61 ± 4.95
Yunnaneic acid B	1	105.75 ± 2.60	105.53 ± 5.71	101.95 ± 7.57
	5	105.95 ± 7.73	104.94 ± 6.73	100.86 ± 5.03
	50	115.62 ± 9.48	113.46 ± 11.30	114.31 ± 12.63
Rosmarinic acid	1	103.53 ± 2.68	103.84 ± 5.93	106.60 ± 7.31
	5	105.01 ± 5.40	106.99 ± 5.54	104.91 ± 9.44
	50	103.82 ± 1.77	105.02 ± 6.58	110.89 ± 12.36
Argatroban	5	total inhibition		

## Data Availability

Data are contained within the article.

## Abbreviations

The following abbreviations are used in this manuscript:

5-LOX	5-lipoxygenase
AChE	acetylcholinesterase
ASC speck	the apoptosis-associated speck-like protein containing a caspase recruitment domain
CA CAD	caffeic acid charged aerosol detector
CFF	clot formation and fibrinolysis assay
Con A	concanavalin A
COX-2	cyclooxygenase-2
DEX	dexamethasone
DPPH^•^	2,2-diphenyl-1-picrylhydrazyl radical
Erk	extracellular signal-regulated kinase
HaCaT cells	human epidermal keratinocyte cell line
HPLC/ESI-MS/MS	high performance liquid chromatography–electrospray ionization tandem mass spectrometry
IFN-γ	interferon-gamma
IL-18	interleukin 18
IL-1β	interleukin 1β
IL-6	interleukin 6
IL-8	interleukin 8
JNK	(c-Jun N-terminal) kinase
LPS	lipopolysaccharide
MAPK	the mitogen-activated protein kinase
MTT	3-(4,5-dimethylthiazol-2-yl)-2,5-diphenyltetrazolium bromide
NF-κB	the nuclear factor kappa-light-chain-enhancer of activated B cells
NHDF	normal human dermal fibroblasts
NLRP3	the NLR family pyrin domain-containing 3 (NLRP3)
NMR	nuclear magnetic resonance
NLRs	nucleotide-binding and oligomerization domain (NOD)-like receptors
p38 MAP kinases	p38 mitogen-activated protein kinases
PBMCs	peripheral blood mononuclear cells
PGE_2_	prostaglandin E2
PRRs	the pattern recognition receptors
RA	rosmarinic acid
SA H	salvianolic acid H
STAT1	signal transducers and activators of transcription 1
STAT3	signal transducers and activators of transcription 3
TF	tissue factor
THP1-ASC-GFP cells	human leukemia monocytic cells stably expressing the green fluorescent protein tagged ASC
TLRs	Toll-like receptors
TNFα UHPLC	tumor necrosis factor alpha ultra-high-performance liquid chromatography
UroA	urolithin A
YA B	yunnaneic acid B
